# Aerobic Exercise Training, Biological Age, and Mortality in Chronic Heart Failure With Reduced Ejection Fraction

**DOI:** 10.1016/j.jacadv.2025.101659

**Published:** 2025-03-14

**Authors:** Zihao Huang, Xinghao Xu, Yan Leng, Zezhi Ke, Ziyue Tang, Ziyan Fan, Rongling Dai, Xinxue Liao, Xiaodong Zhuang, Qi Liang

**Affiliations:** a Department of Rehabilitation Medicine, The First Affiliated Hospital, Sun Yat-Sen University, Guangzhou, China; b Department of Cardiology, The First Affiliated Hospital, Sun Yat-Sen University, Guangzhou, China; c National Health Commission Key Laboratory of Assisted Circulation (Sun Yat-Sen University), Guangzhou, China; d School of Health Science, Guangdong Pharmaceutical University, Guangzhou, China; e School of Journalism and Communication, Sun Yat-Sen University, Guangzhou, China

**Keywords:** aerobic exercise, biological age, heart failure with reduced ejection fraction, mortality

## Abstract

**Background:**

Among individuals with chronic heart failure with reduced ejection fraction (HFrEF), the predictive value for mortality by biomarker-based biological age (BA) and whether aerobic exercise training (AET) modifies the association are understudied.

**Objectives:**

The authors aimed to investigate the association between BA and mortality among individuals with HFrEF and assess whether AET modifies the association.

**Methods:**

Including participants in HF-ACTION (Heart Failure: A Controlled Trial Investigating Outcomes of Exercise Training), BA acceleration was constructed by the Klemera-Doubal method, using the residual of a linear model of BA and chronological age. The associations between BA and all-cause mortality, cardiovascular death, and all-cause hospitalization were investigated by treating BA acceleration into continuous and quintiles in the overall cohort.

**Results:**

Among the 1,732 individuals, during a median of 31.5 (IQR: 20.7-43.1) months of follow-up, 301 deaths were observed. A 1-SD increase in BA acceleration was associated with a 31% higher risk of all-cause mortality (HR: 1.31; 95% CI: 1.13-1.51), a 31% higher risk of cardiovascular mortality (HR: 1.31; 95% CI: 1.12-1.54), and a 9% higher risk of all-cause hospitalization (HR: 1.09; 95% CI: 1.01-1.17). The association of all-cause mortality was significantly different between treatment arms (*P* interaction = 0.024). BA acceleration was associated with a 53% higher risk of all-cause mortality in usual care (HR: 1.53; 95% CI: 1.25-1.89), but the association was not significant in AET (HR: 1.10; 95% CI: 0.89-1.36).

**Conclusions:**

Among individuals with HFrEF, BA has a good prediction value in HFrEF endpoints. AET may be associated with a reduction in all-cause mortality driven by aging.

Heart failure (HF) is a global health pandemic in the progressively aging world population, affecting approximately 64.3 million people worldwide over the past decade, with nearly 50%, classified as HF with reduced ejection fraction (HFrEF).^1^^,^^2^ Aging is a major determinant risk factor for HF, but chronological age only refers to the passage of time.^3^^,^^4^ Given that age-related changes accumulate at hierarchical levels, biological age (BA) has emerged as a more precise indicator of life expectancy, better capturing individual heterogeneity.^4^ To date, several aging measures have been proposed, including epigenetic clocks, clinical biomarker-based BA (eg, Klemera-Doubal method BA [KDM-BA]), and functional age (eg, frailty index).^4^ Biomarker-based BA is cost-effective, making it feasible for population-based studies, and has demonstrated a good performance in predicting risk of mortality in the general population.^5^

Aerobic exercise training (AET) is a well-established nonpharmacological treatment for HFrEF and has been endorsed with a “Class I/Level A” recommendation based on robust evidence in international guidelines.^6^^,^^7^ The HF-ACTION (Heart Failure: A Controlled Trial Investigating Outcomes of Exercise Training) trial, the largest study to date on AET in individuals with HFrEF, demonstrated AET significantly improved health-related outcomes apart from all-cause mortality or hospitalization when comparing to usual care.^8^ Subsequent studies proposed exercise-improved aging-related biomarkers, and the health-related benefits from AET may be more remarkable among individuals with HFrEF and older in chronological age or frailty.^9^, ^10^, ^11^ However, aging is a complex and multifactorial process, and recent measures may capture distinct facets of aging.^4^^,^^12^ Frailty is usually used to describe the aging process in the elderly population, while BA describes an individual age from adulthood.^12^ To date, among individuals with HFrEF, the relationship between biomarker-based BA and the risk of mortality and whether this association is modified by AET remain unclear. Therefore, this study aims to establish biomarker-based BA using KDM and investigate the association between BA and AET with mortality by conducting a post hoc secondary analysis of the HF-ACTION trial among the individuals with HFrEF.

## Methods

### Study design and participants

This study was a post hoc analysis of the HF-ACTION trial (NCT00047437). Deidentified, publicly available data from the HF-ACTION trial was obtained from the National Heart, Lung, and Blood Institute's Biological Specimen and Data Repository Information Coordinating Center. The detailed design, randomization and masking, protocol, and primary trial results of the HF-ACTION trial have been published previously.^8^^,^^13^ Briefly, HF-ACTION was a multicenter, randomized controlled trial that investigated the clinical benefits of AET lasting for 3 to 6 months, compared with usual care in individuals with HFrEF (ejection fraction ≤35% and NYHA functional class II-IV).^13^ The eligibility and exclusion criteria appear in Supplemental Methods.

For the present analysis, 2,130 participants consented, completed follow-up, and had available data in the Biologic Specimen and Data Repository Information Coordinating Center (Supplemental Figure 1). Biomarkers for BA assessments were measured at the baseline and BA calculations were available in 1,918 participants. Participants with missing covariates (186 participants) were excluded. Overall, 1,732 participants were included in the analysis and have similar baseline characteristics as those excluded (Supplemental Table 1).

### Study intervention: aerobic exercise training

Participants in the AET group participated in a 36-session structured, supervised program utilizing cycling or treadmill-based aerobic exercise, with a goal of 3 sessions per week. They were allowed up to 6 months to complete the 36 sessions. After 18 supervised sessions, the program was supplemented with home exercise, transitioning entirely to home-based exercise upon completion of the 36 sessions. The exercise regimen began at 60% heart rate reserve for the initial 6 supervised sessions, lasting 15 to 30 minutes, and progressively increased to 60% to 70% heart rate reserve for 30 to 35 minutes for the remaining supervised sessions and the home-based exercise phase. Both groups, including their families, received self-management education according to the American College of Cardiology/American Heart Association guidelines, including counseling to exercise at moderate intensity for at least 30 minutes per day or as tolerated on most days of the week. Participants in the usual care group did not receive formal written or verbal exercise prescriptions.

### Biological age assessment

BA was constructed based on the clinical biomarkers and anthropometric measurement using the KDM, a validated approach for predicting age-related health outcomes.^5^^,^^14^ The biomarkers were chosen based on their relevance to the aging process, availability in the data sets, and statistical significance and strength of their correlations with chronological age.^15^ In this study, 11 biomarkers were available for BA construction in HF-ACTION. For variables not normally distributed, Spearman correlations and logarithmic transformations were applied when calculating correlation coefficients. Biomarkers significantly correlated with chronological age (|r| > 0.1) were retained, excluding low-density lipoprotein cholesterol due to its high correlation with total cholesterol (r = 0.84) (Supplemental Figure 2).^16^ Finally, 7 biomarkers were selected: body mass index, systolic blood pressure, diastolic blood pressure, total cholesterol, natural logarithmic transformation of creatinine, blood urea nitrogen, and glycated hemoglobin.

The KDM-BA was estimated separately in men and women by the R package “BioAge.” KDM-BA was derived from a series of regressions of individual biomarkers on chronological age in the reference population (details in Supplemental Methods and Supplemental Figures 3 and 4).^17^ Nonpregnant individuals aged 30 to 75 years from the National Health and Nutrition Examination Survey III served as the reference population. To quantify differences in BA among participants, BA acceleration was derived from the residual of a linear model of BA and chronological age. BA and BA acceleration were standardized to have a mean value of 0 and a SD of 1 for continuous analysis, and BA acceleration was divided into quintiles for dose-response analysis.

### Primary and secondary outcomes

The primary outcome was risk of all-cause mortality, and the secondary outcomes included risk of cardiovascular mortality and all-cause hospitalization. Participants were followed up for death for a median of 31.5 months. Outcomes for each patient were assessed by collecting the hospital bills for all hospitalizations and were adjudicated by a clinical endpoint committee blinded to treatment assignment.

### Statistical analysis

Descriptive statistics were used to describe baseline characteristics. Continuous variables are reported as mean ± SD or median (IQR) according to normal distribution, and categorical variables are reported as number and percentage. Baseline characteristics of the study participants were compared across BA acceleration quintiles and treatment arms. The unadjusted risk of the primary outcome was compared across BA acceleration quintiles and between treatment arms using cumulative incidence curves and log-rank tests. Multivariable Cox proportional hazard models were constructed to evaluate the adjusted association of BA and BA acceleration with the risk of primary and secondary outcomes. Models estimating the associations were constructed for the treatment arm and categorical (the first quintile indicating the least BA acceleration as reference) and continuous measures of BA, respectively. Two multivariable models were built to adjust for the potential confounders. Model 1 was adjusted for age, sex, and race at baseline. Model 2 was additionally adjusted for frailty index, smoking status, education, left ventricular ejection fraction, baseline EuroQoL, history of arterial fibrillation or flutter, history of chronic obstructive pulmonary disease, hypertension, six-minute walk distance, renal dysfunction, moderate-to-vigorous physical activity, use of digoxin, use of angiotensin II receptor blocker, and treatment arm at baseline. The association between BA acceleration quintiles and outcomes stratified by treatment arm was further assessed by Cox regression. The proportional hazards assumption was examined by including variables in model 2 (Supplemental Table 2). Restricted cubic splines with 3 knots (10th, 50th, and 90th) were constructed to evaluate the association of BA with the risk of each outcome, stratified by treatment arm. The potential mediating effects of BA on the associations of AET, physical activity, and all-cause mortality were estimated by the mediation model (R package “mediation”).

Multiplicative and additive interaction analyses were performed to investigate whether death induced by BA was modified by treatment arm. The multiplicative interaction was assessed by including a multiplicative interaction term (treatment arm × BA) in model 2. Interaction tests were performed for both continuous and categorical measures of BA. To assess additive interaction, the participants with a mean BA and usual care were set as the reference. The relative excess risk due to interaction and the attributable proportion were assessed.

Sensitivity analyses were conducted by excluding the participants who died or experienced all-cause hospitalization during the intervention period (3-6 months). Some functions in the KDM-BA calculation used the reference population age range from 30 to 75 years.^17^ Sensitivity analyses were also performed by restricting the participants' age consistent with that in the reference population and adapting the PhenoAge method to calculate BA. To investigate the potential sex difference in HFrEF, a subgroup analysis stratified by sex was estimated. A 2-sided *P* < 0.05 was considered statistically significant, and all statistical analyses were performed with R version 4.2.2.

## Results

### Study population

The baseline information of participants with HFrEF according to the treatment arm, BA, or BA acceleration is presented in Table 1 and Supplemental Table 3. This study included 1,732 participants (mean age, 58.8 ± 13.0 years; 27.3% women; 33.4% Black), with 861 treated by AET (Supplemental Table 1). After stratifying BA acceleration quintiles, those participants with higher BA acceleration had a higher burden of comorbidities with worse cardiometabolic situations and physical and renal functions.

**Table 1:** Baseline Characteristics of Study Participants Stratified by Treatment Arms

	Control Group (N = 871)	Exercise Group (N = 861)	*P* Value
Age, y	59.0 ± 13.0	58.6 (12.4)	0.548
Female	220 (25.3)	252 (29.3)	0.069
Race			0.561
Black	283 (32.5)	295 (34.3)	
White	546 (62.7)	519 (60.3)	
Others	42 (4.8)	47 (5.5)	
Education			0.121
Less than high school	110 (12.6)	101 (11.7)	
High school graduate or equivalent	258 (29.6)	221 (25.7)	
Some college	240 (27.6)	228 (26.5)	
Associate degree/diploma program	70 (8.0)	82 (9.5)	
College graduates	119 (13.7)	153 (17.8)	
Completed graduate school	74 (8.5)	76 (8.8)	
Smoking status			0.865
Never	316 (36.3)	316 (36.7)	
Current	149 (17.1)	139 (16.1)	
Former	406 (46.6)	406 (47.2)	
BMI, kg/m^2^	29.8 (25.8-35.3)	29.8 (25.8-34.9)	0.654
SBP, mm Hg	112.0 (100.0-126.0)	110.0 (100.0-126.0)	0.504
DBP, mm Hg	70.0 (60.0-80.0)	70.0 (60.0-78.0)	0.882
Creatinine, mg/dL	1.2 (1.0-1.5)	1.2 (1.0-1.5)	0.115
BUN, mg/dL	21.0 (15.0-29.0)	20.0 (15.0-28.0)	0.624
HbA1c, %	13.5 (12.5-14.6)	13.4 (12.2-14.6)	0.198
Total cholesterol, mg/dL	162.0 (139.0-188.0)	163.0 (135.0-191.0)	0.658
LVEF, %	25.3 (7.4)	25.1 (7.7)	0.689
MVPA, mins/wk	0.0 (0.0-60.0)	0.0 (0.0-40.0)	0.347
EuroQoL	65.6 (19.8)	65.3 (18.1)	0.771
Baseline 6MWD, m	362.1 (106.2)	366.3 (98.2)	0.395
Hypertension	520 (59.7)	535 (62.1)	0.322
History of AF	191 (21.9)	182 (21.1)	0.733
COPD	96 (11.0)	101 (11.7)	0.697
Renal dysfunction	11 (1.3)	14 (1.6)	0.666
ARB medication	190 (21.8)	219 (25.4)	0.086
Digoxin use	414 (47.5)	385 (44.7)	0.260
Frailty index	0.25 (0.11)	0.25 (0.10)	0.982
KDM-BA	54.6 (22.6)	53.1 (22.1)	0.178
KDM-BA advance	0.6 (18.7)	−0.6 (18.9)	0.224

Values are mean ± SD or median (IQR).

6MWD = six-minute walk distance; AF = arterial fibrillation or flutter; ARB = angiotensin II receptor blocker; BMI = body mass index; BUN = blood urea nitrogen; COPD = chronic obstructive pulmonary disease; DBP = diastolic blood pressure; HbA1c = glycated hemoglobin; KDM-BA = Klemera-Doubal method biological age; LVEF = left ventricular ejection fraction; MVPA = moderate-to-vigorous physical activity; SBP = systolic blood pressure.

### Biological age and outcomes

During a median follow-up of 31.5 (IQR: 20.7-43.1) months, death was observed in 301 participants. A 1-SD increase in BA (HR: 1.39; 95% CI: 1.22-1.58) and BA acceleration (HR: 1.24; 95% CI: 1.10-1.41) was associated with a significantly higher risk of all-cause mortality. Compared with the participants with the lowest BA acceleration, those in the highest quintile were associated with an increased risk of all-cause mortality (HR: 1.81; 95% CI: 1.23-2.66) (Table 2, Supplemental Figure 5). Similar significant associations were also observed in cardiovascular death and all-cause hospitalization (Supplemental Table 4, Supplemental Figure 5).

**Table 2:** Association and Interaction of Treatment Arms and Biological Age With All-Cause Mortality

Variables	Events, n/N (%)	Model 1	*P* Value	Model 2	*P* Value	*P*_*interaction*_^a^
BA, per 1-SD increase^b^	301/1732 (17.4)	1.47 (1.32-1.64)	<0.001	1.39 (1.22-1.58)	<0.001	0.022
BA acceleration, per 1-SD increase	301/1732 (17.4)	1.26 (1.14-1.41)	<0.001	1.24 (1.10-1.41)	0.001	0.033
Quintile 1	44/347 (12.7)	1.00 (Reference)	-	1.00 (Reference)	-	0.022
Quintile 2	57/346 (16.5)	1.36 (0.92-2.02)	0.125	1.38 (0.93-2.05)	0.110	
Quintile 3	52/346 (15.0)	1.25 (0.83-1.87)	0.282	1.27 (0.84-1.89)	0.254	
Quintile 4	65/346 (18.8)	1.56 (1.06-2.29)	0.023	1.48 (1.00-2.18)	0.049	
Quintile 5	83/347 (23.9)	1.99 (1.38-2.88)	<0.001	1.81 (1.23-2.66)	0.003	

Model 1 is adjusted by age, race, and sex. Model 2 is adjusted by the covariates in model 1 plus frailty index, smoking status, education, left ventricular ejection fraction, baseline EuroQoL, history of arterial fibrillation or flutter, history of chronic obstructive pulmonary disease, hypertension, six-minute walk distance, renal dysfunction, physical activity, use of digoxin, use of angiotensin II receptor blocker, and treatment arm.

BA = biological age.

a The interaction effect between treatment arm and biological age for the risk of all-cause death is assessed by including a multiplicative interaction term (treatment arm × biological age) in model 2.

b Age was not adjusted in model 1 and model 2.

### AET modified BA and outcomes

**Figure 1:**
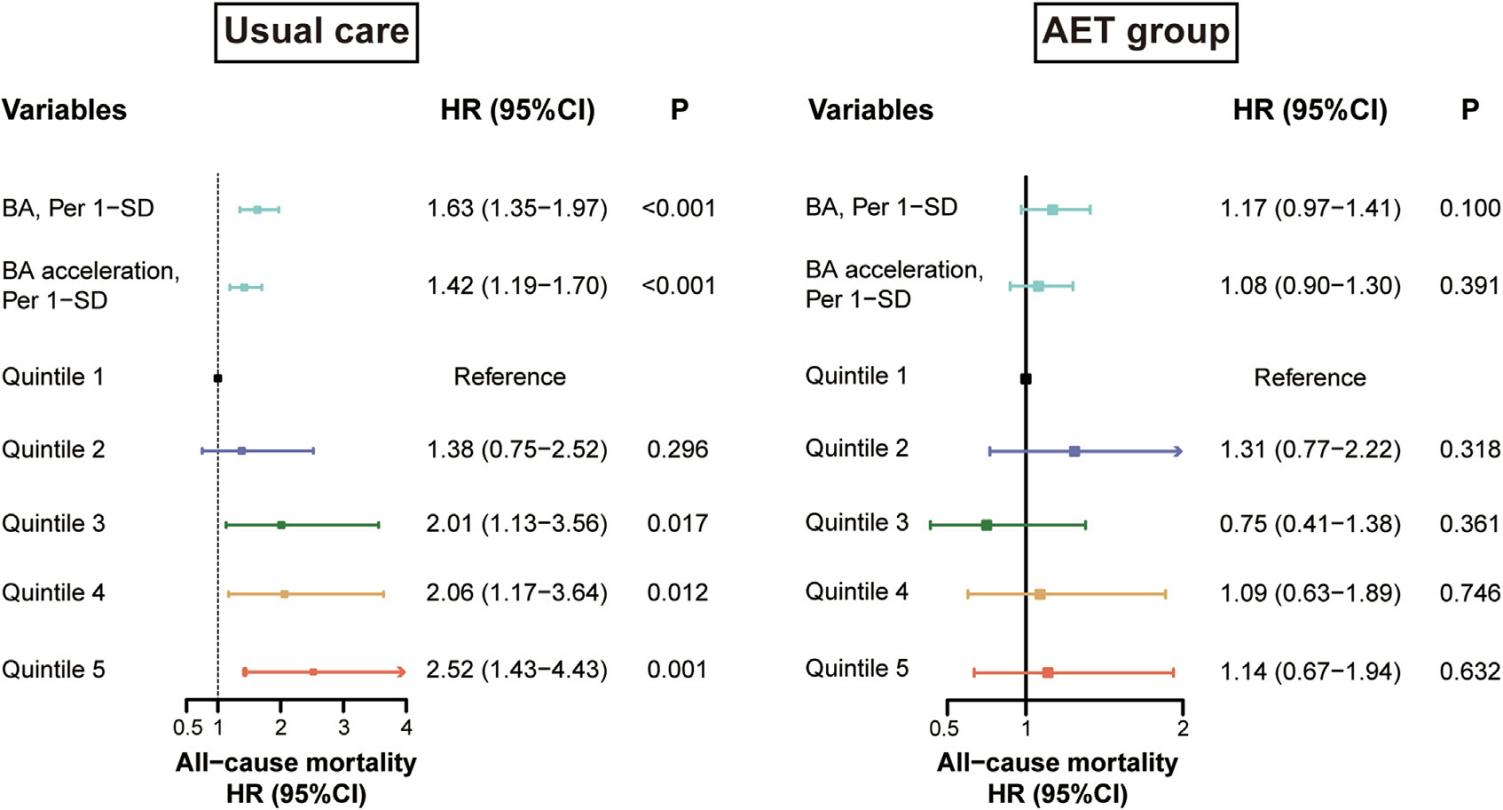
**Association Between Klemera-Doubal Method Biological Age and All-Cause Mortality Stratified by Treatment Arms** Stratified Cox models were constructed for participants under aerobic exercise training (AET) and usual care separately for all-cause mortality with adjustment for the same covariates. Adjusted covariates include age, race, sex, smoking status, education, left ventricular ejection fraction, baseline EuroQoL, history of arterial fibrillation or flutter, history of chronic obstructive pulmonary disease, hypertension, six-minute walk distance, renal dysfunction, physical activity, use of digoxin, and use of angiotensin II receptor blocker. BA = biological age.

**Figure 2:**
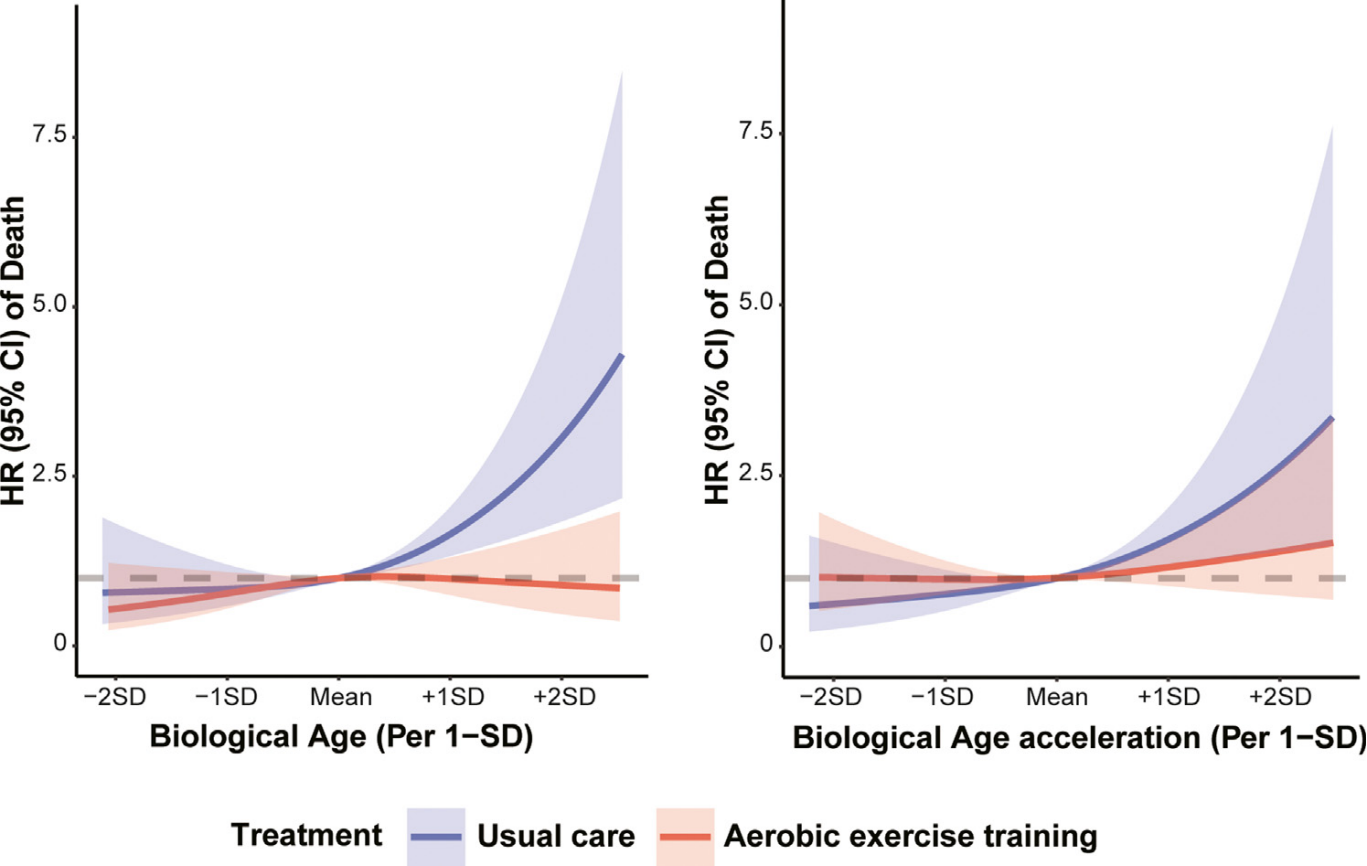
**The Best Fitting Models for Relationships of Klemera-Doubal Method Biological Age With All-Cause Mortality Stratified by Treatment Arms** The solid line indicates the point estimation, ribbons indicate the 95% CIs, and the gray dashed line indicates the reference line (y = 1). Restricted cubic spline regression models were constructed by three knots (10th, 50th, and 90th) for participants in aerobic exercise training and usual care separately for risk of all-cause and cardiovascular death with adjustment for the same covariates. Adjusted covariates include race, sex, smoking status, education, left ventricular ejection fraction, baseline EuroQoL, history of arterial fibrillation or flutter, history of chronic obstructive pulmonary disease, hypertension, six-minute walk distance, renal dysfunction, physical activity, use of digoxin, and use of angiotensin II receptor blocker. Age was additionally adjusted in models of biological age acceleration.

The association of BA (Table 2) (*P* interaction = 0.022) and BA acceleration (*P* interaction = 0.033) with the risk of all-cause mortality significantly differed between those with usual care or AET. In usual care, BA acceleration (per 1 SD; HR: 1.42; 95% CI: 1.19-1.70) was associated with a higher risk of all-cause mortality (Figure 1). However, the relationship between BA acceleration (per 1 SD; HR: 1.08; 95% CI: 0.90-1.30) and the risk of all-cause death was not significant in the participants in the AET group. BA and BA acceleration were associated with an exponential increase in risk of all-cause mortality when they exceeded the means in usual care, but the relationship was suppressed when treated by AET (Figure 2). Similar tendency was observed when stratifying BA/BA acceleration into quintiles (Figure 1, Supplemental Figure 6). A significant additive interaction was observed between AET and BA/BA acceleration on the risk of all-cause mortality (Table 3). The relative excess risk due to interaction in BA acceleration was −0.30 (95% CI: −0.58 to −0.02), and the attributable proportion to the additive interaction was −27% (95% CI: −61% to −5%). These interactions were not significant among the secondary outcomes (Supplemental Table 4, Supplemental Figures 6 and 7).

**Table 3:** Attributing Effects to Additive Interaction Between Increased Biological Aging and Exercise Training on Risk of All-Cause Death

	All-Cause Mortality	
	BA^a^	BA Acceleration
Main effects		
Per 1-SD increase	1.57 (1.33-1.87)	1.40 (1.19-1.65)
AET	1.04 (0.82-1.33)	1.00 (0.79-1.27)
Joint effect	1.27 (0.96-1.67)	1.11 (0.84-1.46)
RERI	−0.35 (−0.68 to −0.04)	−0.30 (−0.58 to −0.02)
AP	−0.28 (−0.61 to −0.06)	−0.27 (−0.61 to −0.05)

Values are HR (95% CI). Models are adjusted for age, race, sex, frailty index, smoking status, education, left ventricular ejection fraction, baseline EuroQoL, history of arterial fibrillation or flutter, history of chronic obstructive pulmonary disease, hypertension, 6-minute walk distance, renal dysfunction, physical activity, use of digoxin, and use of angiotensin II receptor blocker.

AET = aerobic exercise training; AP = attributable proportion; BA = biological age; RERI = relative excess risk due to interaction.

a Age was not adjusted.

Compared to the usual care, the direct effect of AET was nonsignificant with a lower risk of all-cause mortality (Supplemental Table 5), but the improving tendency was more evident among the individuals with higher BA acceleration. Neither the AET nor baseline moderate-to-vigorous physical activity volume was mediated on the relationship of BA/BA acceleration and all-cause death (Supplemental Figure 8).

### Sensitivity analysis

The associations of BA and BA acceleration with risk of all-cause mortality were still significantly different from treatment arms after excluding the participants who died or experienced all-cause hospitalization during the first 3 to 6 months (Supplemental Tables 6 and 7). Similar results were observed when restricting the participants' age consistent with the reference population calculating KDM-BA (Supplemental Table 8) and involving the PhenoAge method for BA calculation (Supplemental Table 9). Consistent results were also shown in the subgroup analysis stratified by sex (Supplemental Table 10).

## Discussion

This post hoc secondary analysis of the HF-ACTION trial was performed in individuals with HFrEF to determine the association of BA and AET with mortality (Central Illustration). This study found that increased KDM-BA was associated with a higher risk of all-cause mortality, cardiovascular mortality, and all-cause hospitalization in HFrEF. The association of BA with all-cause mortality was significant in usual care, rather than in AET, and the survival benefit may be more pronounced among those with advanced BA. These findings highlight the contribution of AET in mitigating the adverse effects of advanced BA among individuals with HFrEF.

**Central Illustration:**
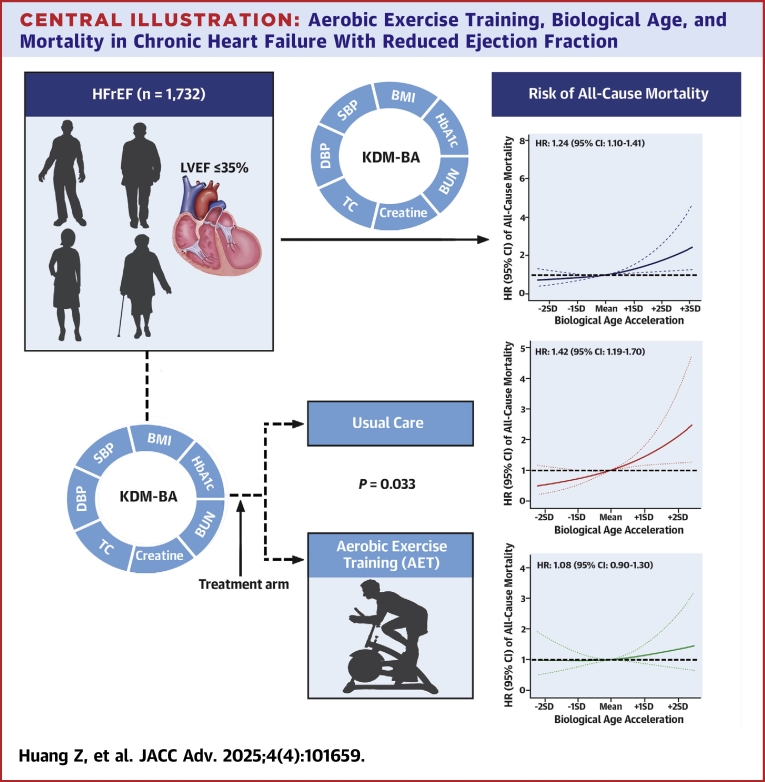
A**erobic Exercise Training, Biological Age, and Mortality in Chronic Heart Failure With Reduced Ejection Fraction** Biological age (BA) was constructed and analyzed for the risk of all-cause mortality among the 1,732 heart failure individuals. The associations stratified by the treatment arm (aerobic exercise training vs usual care) were further investigated. The 3 graphs show the association of BA and risk of all-cause mortality referring to the mean BA acceleration and further stratified by aerobic exercise training and usual care. The *P* for interaction was computed using multivariable Cox regression by including a multiplicative interaction term (treatment arm × BA acceleration) and adjusting age, race, sex, frailty index, smoking status, education, left ventricular ejection fraction, baseline EuroQoL, history of arterial fibrillation or flutter, history of chronic obstructive pulmonary disease, hypertension, six-minute walk distance, renal dysfunction, physical activity, use of digoxin, and use of angiotensin II receptor blocker. These data show that among the individuals with chronic heart failure with reduced ejection fraction, the risk of all-cause mortality increased over BA acceleration and aerobic exercise training may significantly alleviate the association.

Biomarker-based BA is considered a cost-effective aging indicator and a well-performed predictor of mortality in general population.^4^^,^^12^^,^^18^ AET is widely recommended in clinical guidelines for its potential to improve biomarkers related to BA and extend longevity across various populations.^7^^,^^11^^,^^19^ However, the evidence regarding its benefits in HFrEF remains inconsistent, and the HF-ACTION trial, conducted to evaluate effectiveness of AET on mortality with adequate power in a large sample, disclosed a nonsignificant difference in HF endpoints when comparing AET to usual care.^8^^,^^10^^,^^20^ Subsequent studies did not draw a definitive conclusion of the survival benefits from AET among HFrEF.^10^^,^^20^ Recent post hoc analyses of large-scale clinical trials, including HF-ACTION and Rehabilitation Therapy in Older Acute Heart Failure Patients (REHAB-HF), disclosed the AET-related benefits may be more pronounced in individuals with frailty.^9^^,^^21^ The present study also found that AET was associated with a lower risk of all-cause mortality driven by aging, with the survival benefits being more pronounced in those with advanced BA.

Consistent with prior studies, KDM-BA is associated with an increased risk of mortality and hospitalization, and KDM-BA may provide more comprehensive information on aging process compared to chronological age alone.^5^ In the current study, KDM-BA served the same role in predicting all-cause mortality, cardiovascular mortality, and all-cause hospitalization among individuals with HFrEF. However, the joint association of AET and aging with cardiovascular mortality or all-cause hospitalization was nonsignificant. Similar to previous findings, the efficacy of AET on HF endpoints varies across individuals' chronological age and frailty status.^9^^,^^10^^,^^21^ The conflicting results regarding AET's benefits on survival and hospitalization may be partially attributed to the different aging measures, which may capture distinct aspects of aging and are associated with different endpoints.^4^^,^^22^ Frailty describes the individuals' functional incapacity unidirectionally in the elderly, while KDM-BA indicates how modifiable biomarkers modulate the rate of aging from adulthood and consequently the longevity.^4^^,^^9^^,^^12^^,^^18^ The direct or mediating effects of AET and baseline physical activity were nonsignificant. One potential explanation is the timing of measurements for these parameters and the lag in their effects. In the HF-ACTION trial, physical activity and parameters associated with KDM-BA were measured at baseline, with randomization assigning participants to AET or usual care, while endpoints were assessed prospectively. This design limited further exploration of the relationships among these parameters.

The reasons for survival benefits attained from AET varying in BA are multifaceted.^3^^,^^11^ Prior reviews have summarized the mechanisms from exercise, including improved metabolic functions, reduced sarcopenia, and alleviated oxidative stress.^3^^,^^11^ However, the specific mechanisms by which AET attenuates the risk of all-cause mortality driven by KDM-BA in individuals with HFrEF were understudied. This population often exhibits worse metabolic function and more pronounced muscle degeneration, which can further impair already weakened skeletal muscle biology due to HFrEF.^23^^,^^24^ Given that many exercise-induced benefits are driven by physiological changes within skeletal muscle,^11^ the degenerative effects of aging and disease may be mitigated through AET. It is possible that greater baseline aging-related impairments among individuals with HFrEF may have provided greater potential for survival benefits.^21^^,^^25^ Nevertheless, due to limited regenerative capacity in myocardial cells, HFrEF is often accompanied by unmodifiable and irreversible ventricular remodeling.^26^ Exercise, as a behavioral intervention, may help alleviate the burden of all-cause mortality by addressing modifiable aging-related factors such as oxidative stress, blood pressure, and skeletal muscle metabolism, rather than having profound impacts on reversing the inherently poor ventricular function.^11^^,^^21^ This may also explain the modest survival benefits and the negative results observed in prior studies.

This study highlights the value of endpoint prediction of KDM-BA in HFrEF and AET in reducing all-cause mortality caused by biological aging. First, KDM-BA serves as an effective and biomarker-based aging predictor for HF endpoints in HFrEF, offering a more precise stratification tool compared to chronological age alone. The biomarker-based KDM-BA may enhance patient stratification in clinical practice. Second, AET and the maintenance of regular exercise behavior are crucial even for individuals with HFrEF, particularly for those experiencing advanced biological aging, who may derive additional survival benefits. This insight could assist clinicians in tailoring exercise prescriptions and emphasizing the exercise-induced benefits for patients identified with advanced BA. Third, future clinical trials targeting HFrEF may consider age-based exercise interventions quantifying the volume or duration that account for both chronological age and BA to clarify the causal relationships among exercise, aging, and survival benefits. Finally, this study provides new evidence supporting current guideline recommendations on exercise-based cardiac rehabilitation as a potential means to improve survival outcomes.^6^^,^^7^

### Strengths and limitations

The present study has several strengths, including the construction of an aging indicator based on clinical biomarkers, the inclusion of individuals with profound reductions in ejection fraction (<35%), and the use of data from a multicenter randomized controlled trial with a substantial sample size and long follow-up period. However, this study also has several limitations. First, as a post hoc analysis of the HF-ACTION trial, the results should be interpreted with caution, as the analysis may not be adequately powered to fully determine the effects of AET on survival benefits in individuals with HFrEF and advanced BA. To address this, we categorized BA into both continuous and categorical variables and combined them with different sensitivity analyses in our primary results. Second, participants in HF-ACTION were relatively stable and able to exercise at baseline, which may limit the generalizability of our findings to a broader HFrEF population. Third, the primary HF-ACTION trial assessed a limited number of clinical biomarkers at baseline, which restricts the possibility to achieve a more precise estimation of KDM-BA, investigates the direct or mediating effects of AET or physical activity on BA, and observes a longitudinal change of BA. Nonetheless, the present KDM-BA still shows a good correlation with chronological age, and the association with all-cause mortality is robust. Fourth, similar to other post hoc analyses, the influence of dietary intake, longitudinal exercise volume, perimenopausal, or other unmeasured confounders could not be excluded, but we have excluded the key confounders.

## Conclusions

Among individuals with HFrEF, KDM-BA constructed by clinical biomarkers has a good predictive value for mortality and hospitalization compared to chronological age alone. AET may be associated with a reduction in all-cause mortality driven by aging.

## Funding support and author disclosures

The authors have reported that they have no relationships relevant to the contents of this paper to disclose.
