# Supplementary material for: Aerobic Exercise Training, Biological Age, and Mortality in Chronic Heart Failure With Reduced Ejection Fraction
Source: JACC Adv. 2025 Mar 14;4(4):101659. doi: 10.1016/j.jacadv.2025.101659 (PMC11937671; doi:10.1016/j.jacadv.2025.101659)
Supplement: Supplemental data [file mmc1.docx]

**SUPPLEMENTAL APPENDIX**

Supplemental Methods

Eligibility and exclusion criteria

In the primary design of Heart Failure: A Controlled Trial Investigating Outcomes of Exercise Training, the eligibility criteria included individuals with an LVEF of ≤ 35%, and NYHA class II-IV heart failure (HF) persisting for at least 3 months despite a minimum of 6 weeks of treatment. Participants were required to be on optimal HF therapy at stable doses for 6 weeks prior to enrolment, or have a documented reason for variation, such as intolerance, contraindications, patient preference, or the judgment of their personal physician. Additionally, participants had to be deemed sufficiently stable by the investigator to begin an exercise program.^1^

Exclusion criteria included being under 18 years of age, having comorbid conditions or behavioural limitations that would interfere with exercise training or prevent completion of a 1-year exercise program, or experiencing a major cardiovascular event or procedure, including implantable cardioverter-defibrillator (ICD) placement or cardiac resynchronization, within the previous 6 weeks. Participants were also excluded if they had a planned cardiovascular procedure or hospitalization (within 6 months for ICD or cardiac resynchronization therapy), were currently pregnant or planning to become pregnant within the next year, expected to receive a cardiac transplant within the next 6 months, had HF secondary to significant uncorrected primary valvular disease (except mitral regurgitation secondary to LV dysfunction), HF due to congenital heart disease or obstructive cardiomyopathy, or had engaged in regular exercise training (more than once per week at moderate-to-vigorous intensity) within the previous 6 weeks. Additional exclusions included the use of fixed-rate pacemakers, pacemakers unable to attain target heart rates, or ICDs with heart rate limits set below the target for exercise training, as well as exercise testing results that would preclude safe exercise training or co-enrolment in a clinical trial not approved for co-participation.

Construction of biological age

Calculation of biological age (BA) under Klemera and Doubal’s method (KDM) and BA acceleration under Klemera and Doubal’s method^2^

$KDM-BA=\frac{\sum_{j=1}^{m} \left( x_{j}-q_{j} \right)\left( \frac{k_{j}}{s_{j}^{2}} \right)+\frac{\mathrm{CA}}{s_{\mathrm{BA}}^{2}}}{\sum_{j=1}^{m} \left( \frac{k_{j}}{s_{j}^{2}} \right)+\frac{1}{s_{\mathrm{BA}}^{2}}}$ (1.1)

m: the number of principal components;

x_j_: the jth principal components;

q_j_: the intercept of the regression of jth principal components on BA, since BA is unknown, it is replaced by chronological age (CA), the same as below;

k_j_: the slope of the regression of jth principal components on BA;

s_j_: the root mean square error of the regression of jth principal components on BA;

s_BA_: the root mean square error of the regression of CA on BA, and it can be replaced by the root mean square error of the regression of all principal components on CA account of unknown BA.

KDM-BA acceleration= KDM-BA – β * CA (1.2)

The residual of a linear model between KDM-BA and CA is the KDM-BA acceleration. The correlation coefficients of KDM-BA and BA acceleration with CA was calculated. A good correlation between KDM-BA and CA was observed, and a non-significant correlation was observed between BA acceleration and CA (Figure S3-S4).

Covariates

Demographic and clinical characteristics were assessed before randomization by prior published standard protocols.^1^ Age, race, sex (male/female), education level, and smoking status were collected via self-report questionnaires. Race was categorized as Black, White, or Others, with the "Others" category encompassing American Indian/Alaska Native, Asian, Native Hawaiian/Other Pacific Islander, and other races, collapsed due to small sample sizes. Education level was classified into less than high school, high school graduate or equivalent, some college, associate degree/diploma program, college graduate, and completed graduate school. Blood samples for laboratory measurements were obtained by trained staff before randomization. Left ventricular ejection fraction was measured via echocardiography after the patient had maintained a stable dose of any therapy or intervention for at least six weeks. Health-related quality of life was assessed using the EuroQoL thermometer response (0 to 100), and moderate-to-vigorous physical activity (minutes/week) was measured using the International Physical Activity Questionnaire at baseline. The six-minute walk test (in meters) was performed at least two hours before or after the exercise test. Medical history (yes/no), including the presence of atrial fibrillation or flutter, chronic obstructive pulmonary disease, hypertension, and medication use (yes/no), including digoxin, angiotensin II receptor blockers, were reviewed by trained staff at the baseline visit. Renal dysfunction was defined as creatinine levels exceeding 3 mg/dL. Frailty index was calculated including the variables on symptoms, signs, disabilities, activity of daily living, self-rated health, and comorbidities based on prior post-hoc analysis. ^3^

**References**

1. Whellan DJ, O'Connor CM, Lee KL, et al. Heart failure and a controlled trial investigating outcomes of exercise training (HF-ACTION): design and rationale. *Am Heart J.* Feb 2007;153(2):201-211. doi: 10.1016/j.ahj.2006.11.007.

2. Kwon D, Belsky DW. A toolkit for quantification of biological age from blood chemistry and organ function test data: BioAge. *Geroscience.* Dec 2021;43(6):2795-2808. doi: 10.1007/s11357-021-00480-5.

3. Pandey A, Segar MW, Singh S, et al. Frailty Status Modifies the Efficacy of Exercise Training Among Patients With Chronic Heart Failure and Reduced Ejection Fraction: An Analysis From the HF-ACTION Trial. *Circulation*. 2022-07-12 2022;146(2):80-90. doi: 10.1161/CIRCULATIONAHA.122.059983.

Supplemental Table 1 Baseline characteristics of study participants of overall cohort

|  | Included (N=1732) | Excluded (N=398) | P |
| --- | --- | --- | --- |
| Age, years | 58.8 (12.7) | 57.6 (12.4) | 0.100 |
| Female, n (%) | 472 (27.3) | 127 (31.9) | 0.072 |
| Race, n (%) |  |  | 0.005 |
| Black | 578 (33.4) | 91 (24.9) |  |
| White | 1065 (61.5) | 257 (70.2) |  |
| Others | 89 (5.1) | 18 (4.9) |  |
| Education, n (%) |  |  | 0.972 |
| Less than high school | 211 (12.2) | 44 (12.5) |  |
| High school graduate or equivalent | 479 (27.7) | 94 (26.8) |  |
| Some college | 468 (27.0) | 101 (28.8) |  |
| Associate degree/diploma program | 152 (8.8) | 32 (9.1) |  |
| College graduates | 272 (15.7) | 53 (15.1) |  |
| Completed graduate school | 150 (8.7) | 27 (7.7) |  |
| Smoking status, n (%) |  |  | 0.197 |
| Never | 632 (36.5) | 151 (38.9) |  |
| Current | 288 (16.6) | 74 (19.1) |  |
| Former | 812 (46.9) | 163 (42.0) |  |
| BMI, kg/m^2^ | 29.8 [25.8, 35.1] | 29.9 [26.2, 35.1] | 0.616 |
| SBP, mmHg | 111.0 [100.0, 126.0] | 110.0 [100.0, 124.0] | 0.625 |
| DBP, mmHg | 70.0 [60.0, 78.0] | 70.0 [60.0, 78.0] | 0.359 |
| Creatinine, mg/dL | 1.2 [1.0, 1.5] | 1.2 [1.0, 1.4] | 0.237 |
| BUN, mg/dL | 21.0 [15.0, 28.0] | 20.0 [15.6, 28.0] | 0.597 |
| HbA1c, % | 13.5 [12.3, 14.6] | 14.0 [12.7, 15.0] | 0.001 |
| Total cholesterol, mg/dL | 162.0 [137.0, 189.0] | 162.0 [137.0, 197.0] | 0.711 |
| LVEF, % | 25.2 (7.5) | 25.2 (7.2) | 0.950 |
| MVPA, mins/week | 0.0 [0.0, 50.5] | 0.0 [0.0, 76.2] | 0.052 |
| EuroQoL | 65.5 (19.0) | 66.4 (18.7) | 0.421 |
| Baseline 6MWD, m | 364.2 (102.3) | 374.0 (108.7) | 0.104 |
| Hypertension, n (%) | 1055 (60.9) | 205 (53.1) | 0.006 |
| History of AF, n (%) | 373 (21.5) | 70 (17.6) | 0.097 |
| COPD, n (%) | 197 (11.4) | 35 (9.2) | 0.265 |
| Renal dysfunction, n (%) | 25 (1.4) | 2 (1.1) | 0.978 |
| ARB medication, n (%) | 409 (23.6) | 84 (21.1) | 0.315 |
| Digoxin use, n (%) | 799 (46.1) | 165 (41.5) | 0.102 |
| Frailty index | 0.25 (0.11) | 0.24 (0.11) | 0.009 |
| KDM-BA | 53.9 (22.4) | 53.0 (19.9) | 0.598 |
| KDM-BA advance | 0.0 (18.8) | 0.0 (17.3) | 0.972 |
| Exercise intervention, n (%) | 861 (49.7) | 199 (50.0) | 0.961 |

Data are presented as mean (SD) or median [interquartile range] if not specified. BMI indicates body mass index; SBP, systolic blood pressure; DBP, diastolic blood pressure; BUN, blood urea nitrogen; HbA1c, glycated hemoglobin; LVEF, left ventricular ejection fraction; MVPA, moderate-to-vigorous physical activity; 6MWD, six-minute walk distance; AF, arterial fibrillation or flutter; COPD, chronic obstructive pulmonary disease; ARB, angiotensin II receptor blocker; and KDM-BA, Klemera-Doubal method biological age.

Supplemental Table 2 Proportional hazards assumption of Cox regression models

| **Variables** | **BA** | | **BA acceleration** | |
| --- | --- | --- | --- | --- |
|  | **ꭓ^2^** | ***P*** | **ꭓ^2^** | ***P*** |
| BA | 0.87 | 0.352 | 0.31 | 0.580 |
| Age | NA | NA | 0.88 | 0.347 |
| Sex | 0.06 | 0.804 | 0.08 | 0.778 |
| Race | 2.80 | 0.247 | 2.83 | 0.242 |
| Smoking status | 1.32 | 0.516 | 1.40 | 0.496 |
| Education | 1.03 | 0.311 | 1.08 | 0.299 |
| LVEF | 0.87 | 0.350 | 0.83 | 0.362 |
| EuroQoL | 1.51 | 0.219 | 1.57 | 0.210 |
| History of AF | 0.56 | 0.453 | 0.56 | 0.452 |
| Digoxin use | 3.33 | 0.068 | 3.32 | 0.068 |
| Renal dysfunction | 0.08 | 0.779 | 0.09 | 0.764 |
| Hypertension | 0.42 | 0.518 | 0.37 | 0.541 |
| Baseline 6MWD | 0.87 | 0.351 | 0.82 | 0.365 |
| MVPA | 2.24 | 0.134 | 2.28 | 0.131 |
| COPD | 0.57 | 0.449 | 0.46 | 0.497 |
| ARB medication | 0.52 | 0.471 | 0.52 | 0.469 |
| Exercise intervention | 0.15 | 0.701 | 0.15 | 0.698 |
| Frailty index | 0.62 | 0.432 | 0.60 | 0.438 |
| Global | 20.19 | 0.383 | 20.24 | 0.443 |

Abbreviations: BMI indicates body mass index; SBP, systolic blood pressure; DBP, diastolic blood pressure; BUN, blood urea nitrogen; HbA1c, glycated hemoglobin; LVEF, left ventricular ejection fraction; MVPA, moderate-to-vigorous physical activity; 6MWD, six-minute walk distance; AF, arterial fibrillation or flutter; COPD, chronic obstructive pulmonary disease; ARB, angiotensin II receptor blocker; and BA, biological age.

Supplemental Table **3 Baseline characteristics of study participants stratified by biological aging acceleration quintiles**

|  | Q1 | Q2 | Q3 | Q4 | Q5 | P |
| --- | --- | --- | --- | --- | --- | --- |
| Age, years | 59.5 (12.2) | 58.8 (11.8) | 57.7 (13.1) | 58.6 (13.4) | 59.2 (13.1) | 0.419 |
| Female, n (%) | 96 (27.7) | 124 (35.8) | 121 (35.0) | 87 (25.1) | 44 (12.7) | <0.001 |
| Race, n (%) |  |  |  |  |  | 0.156 |
| Black | 98 (28.2) | 109 (31.5) | 125 (36.1) | 129 (37.3) | 117 (33.7) |  |
| White | 224 (64.6) | 217 (62.7) | 207 (59.8) | 201 (58.1) | 216 (62.2) |  |
| Others | 25 (7.2) | 20 (5.8) | 14 (4.0) | 16 (4.6) | 14 (4.0) |  |
| Education, n (%) |  |  |  |  |  | 0.514 |
| Less than high school | 41 (11.8) | 35 (10.1) | 43 (12.4) | 46 (13.3) | 46 (13.3) |  |
| High school graduate or equivalent | 98 (28.2) | 99 (28.6) | 91 (26.3) | 94 (27.2) | 97 (28.0) |  |
| Some college | 98 (28.2) | 93 (26.9) | 97 (28.0) | 94 (27.2) | 86 (24.8) |  |
| Associate degree/diploma program | 23 (6.6) | 31 (9.0) | 34 (9.8) | 41 (11.8) | 23 (6.6) |  |
| College graduates | 51 (14.7) | 55 (15.9) | 59 (17.1) | 44 (12.7) | 63 (18.2) |  |
| Completed graduate school | 36 (10.4) | 33 (9.5) | 22 (6.4) | 27 (7.8) | 32 (9.2) |  |
| Smoking status, n (%) |  |  |  |  |  | 0.731 |
| Never | 120 (34.6) | 131 (37.9) | 127 (36.7) | 125 (36.1) | 129 (37.2) |  |
| Current | 67 (19.3) | 49 (14.2) | 64 (18.5) | 56 (16.2) | 52 (15.0) |  |
| Former | 160 (46.1) | 166 (48.0) | 155 (44.8) | 165 (47.7) | 166 (47.8) |  |
| BMI, kg/m^2^ | 28.6 [24.7, 33.5] | 29.2 [25.0, 34.3] | 30.5 [26.6, 35.6] | 30.1 [26.2, 35.8] | 30.3 [26.4, 35.8] | <0.001 |
| SBP, mmHg | 104.0 [94.0, 114.0] | 110.0 [100.0, 120.0] | 112.5 [100.0, 124.0] | 114.0 [104.0, 130.0] | 124.0 [108.5, 140.0] | <0.001 |
| DBP, mmHg | 66.0 [60.0, 75.0] | 68.0 [60.0, 72.0] | 70.0 [62.0, 78.0] | 70.0 [64.0, 80.0] | 72.0 [66.0, 80.0] | <0.001 |
| Creatinine, mg/dL | 1.0 [0.9, 1.1] | 1.0 [0.9, 1.2] | 1.1 [1.0, 1.3] | 1.3 [1.1, 1.5] | 1.7 [1.4, 2.1] | <0.001 |
| BUN, mg/dL | 15.0 [12.0, 21.0] | 17.0 [14.0, 22.0] | 20.0 [15.0, 25.0] | 23.0 [18.0, 31.0] | 33.5 [23.5, 47.0] | <0.001 |
| HbA1c, % | 13.2 [12.2, 13.9] | 13.0 [12.2, 14.2] | 13.5 [12.5, 14.5] | 13.6 [12.6, 14.8] | 13.8 [12.4, 15.0] | <0.001 |
| Total cholesterol, mg/dL | 157.0 [133.0, 182.5] | 164.0 [137.5, 191.0] | 163.5 [138.2, 186.0] | 163.0 [139.0, 197.0] | 164.0 [139.0, 193.0] | 0.116 |
| LVEF, % | 25.5 (7.8) | 25.4 (8.0) | 25.6 (7.9) | 24.5 (7.2) | 25.0 (6.7) | 0.281 |
| MVPA, mins/week | 0.0 [0.0, 45.0] | 0.0 [0.0, 60.0] | 0.0 [0.0, 40.0] | 0.0 [0.0, 37.5] | 0.0 [0.0, 60.0] | 0.928 |
| EuroQoL | 65.7 (18.9) | 66.3 (18.5) | 65.8 (18.8) | 65.1 (18.9) | 64.4 (19.8) | 0.732 |
| Baseline 6MWD, m | 371.8 (93.7) | 370.5 (104.4) | 369.9 (104.4) | 350.5 (103.6) | 358.2 (103.7) | 0.019 |
| Hypertension, n (%) | 186 (53.6) | 177 (51.2) | 196 (56.6) | 235 (67.9) | 261 (75.2) | <0.001 |
| History of AF, n (%) | 57 (16.4) | 76 (22.0) | 71 (20.5) | 78 (22.5) | 91 (26.2) | 0.035 |
| COPD, n (%) | 36 (10.4) | 41 (11.8) | 44 (12.7) | 31 (9.0) | 45 (13.0) | 0.417 |
| Renal dysfunction, n (%) | 0 (0.0) | 0 (0.0) | 0 (0.0) | 0 (0.0) | 25 (7.2) | <0.001 |
| ARB medication, n (%) | 62 (17.9) | 87 (25.1) | 79 (22.8) | 94 (27.2) | 87 (25.1) | 0.045 |
| Digoxin use, n (%) | 161 (46.4) | 159 (46.0) | 157 (45.4) | 160 (46.2) | 162 (46.7) | 0.998 |
| Frailty index | 0.24 (0.10) | 0.23 (0.1) | 0.25 (0.10) | 0.26 (0.11) | 0.28 (0.11) | <0.001 |
| KDM-BA | 29.7 (14.2) | 44.4 (11.6) | 52.0 (12.9) | 62.1 (13.2) | 81.2 (18.3) | <0.001 |
| KDM-BA advance | -24.8 (8.4) | -9.5 (2.8) | -0.9 (2.4) | 8.4 (3.1) | 26.9 (12.7) | <0.001 |
| Exercise intervention, n (%) | 178 (51.3) | 170 (49.1) | 173 (50.0) | 180 (52.0) | 160 (46.1) | 0.567 |

Data are presented as mean (SD) or median [interquartile range] if not specified. BMI indicates body mass index; SBP, systolic blood pressure; DBP, diastolic blood pressure; BUN, blood urea nitrogen; HbA1c, glycated hemoglobin; LVEF, left ventricular ejection fraction; MVPA, moderate-to-vigorous physical activity; 6MWD, six-minute walk distance; AF, arterial fibrillation or flutter; COPD, chronic obstructive pulmonary disease; ARB, angiotensin II receptor blocker; and KDM-BA, Klemera-Doubal method biological age.

Supplemental Table 4 Association between cardiovascular death and all-caused hospitalization with KDM biological age

|  | **Events,**  **n (%)** | **Model 1** | ***P*** | **Model 2** | ***P*** | ^a^***P_interaction_*** |
| --- | --- | --- | --- | --- | --- | --- |
| *Cardiovascular death* | | | | | | |
| ^b^BA, Per 1-SD | 209/1732 (12.1) | 1.54 (1.35-1.75) | <0.001 | 1.45 (1.23-1.71) | <0.001 | 0.045 |
| BA acceleration, per 1-SD | 209/1732 (12.1) | 1.34 (1.18-1.52) | <0.001 | 1.31 (1.12-1.54) | 0.001 | 0.176 |
| Quintile 1 | 26/347 (7.5) | 1 (Reference) | - | 1 (Reference) | - | 0.112 |
| Quintile 2 | 44/346 (12.7) | 1.74 (1.07-2.83) | 0.025 | 1.94 (1.19-3.18) | 0.008 |  |
| Quintile 3 | 35/346 (10.1) | 1.48 (0.89-2.46) | 0.132 | 1.52 (0.92-2.54) | 0.105 |  |
| Quintile 4 | 41/346 (11.8) | 1.79 (1.10-2.94) | 0.020 | 1.73 (1.05-2.86) | 0.032 |  |
| Quintile 5 | 63/347 (18.2) | 2.73 (1.72-4.34) | <0.001 | 2.42 (1.50-3.93) | <0.001 |  |
| *All-caused hospitalization* | | | | | | |
| ^b^BA, Per 1-SD | 969/1732 (55.9) | 1.18 (1.11-1.26) | <0.001 | 1.12 (1.04-1.20) | 0.004 | 0.751 |
| BA acceleration, per 1-SD | 969/1732 (55.9) | 1.13 (1.06-1.20) | <0.001 | 1.09 (1.01-1.17) | 0.025 | 0.172 |
| Quintile 1 | 178/347 (51.3) | 1 (Reference) | - | 1 (Reference) | - | 0.512 |
| Quintile 2 | 195/346 (56.4) | 1.18 (0.96-1.45) | 0.112 | 1.20 (0.98-1.47) | 0.083 |  |
| Quintile 3 | 178/346 (51.4) | 1.08 (0.88-1.33) | 0.473 | 1.05 (0.86-1.30) | 0.623 |  |
| Quintile 4 | 207/346 (59.8) | 1.30 (1.06-1.59) | 0.011 | 1.16 (0.95-1.43) | 0.145 |  |
| Quintile 5 | 211/347 (60.8) | 1.38 (1.13-1.68) | 0.002 | 1.19 (0.96-1.46) | 0.114 |  |

Abbreviations: AET, aerobic exercise training; BA, biological age.

Model 1 is adjusted by age, race and sex. Model 2 is adjusted by the covariates in model 1 plus frailty index, smoking status, education, left ventricular ejection fraction, baseline EuroQoL, history of arterial fibrillation or flutter, history of chronic obstructive pulmonary disease, hypertension, six-minute walk distance, renal dysfunction, physical activity, use of digoxin, use of angiotensin II receptor blocker and treatment arm.

^a^ The interaction effect between treatment arm and biological age for the risk of the all-cause death is assessed by including a multiplicative interaction term (treatment arm × biological age) in model 2.

^b^ Age was not adjusted in model 1 and model 2.

Supplemental Table 5 Joint association of aerobic exercise training and biological age acceleration on heart failure outcomes

| **Variables** | **All-cause mortality** | | **Cardiovascular mortality** | | **All-cause hospitalization** | |
| --- | --- | --- | --- | --- | --- | --- |
|  | **HR (95%CI)** | ***P*** | **HR (95%CI)** | ***P*** | **HR (95%CI)** | ***P*** |
| Quintile 1 | 1.40 (0.77-2.56) | 0.268 | 0.95 (0.44-2.05) | 0.899 | 1.05 (0.78-1.41) | 0.740 |
| Quintile 2 | 1.29 (0.76-2.17) | 0.345 | 1.32 (0.72-2.40) | 0.368 | 0.84 (0.63-1.11) | 0.227 |
| Quintile 3 | 0.59 (0.33-1.04) | 0.067 | 0.59 (0.30-1.18) | 0.137 | 0.77 (0.57-1.04) | 0.083 |
| Quintile 4 | 0.74 (0.45-1.20) | 0.218 | 0.62 (0.33-1.16) | 0.138 | 1.02 (0.77-1.34) | 0.904 |
| Quintile 5 | 0.89 (0.58-1.37) | 0.601 | 0.92 (0.56-1.51) | 0.749 | 0.98 (0.75-1.29) | 0.909 |

Hazard ratio refer to the usual care.

Supplemental Table 6 Association of KDM-BA with death and the interaction with treatment arm by excluding participants died in the first 3 or 6 months.

|  | **Events,**  **n (%)** | **Model 1** | ***P*** | **Model 2** | ***P*** | ^a^***P_interaction_*** |
| --- | --- | --- | --- | --- | --- | --- |
| *Excluding participants died in the first 3 months* | | | | | | |
| ^b^BA, Per 1-SD | 285/1711 (16.7) | 1.49 (1.33-1.66) | <0.001 | 1.42 (1.24-1.63) | <0.001 | 0.034 |
| BA acceleration, per 1-SD | 285/1711 (16.7) | 1.27 (1.13-1.41) | <0.001 | 1.25 (1.10-1.43) | 0.001 | 0.086 |
| Quintile 1 | 42/342 (12.3) | 1 (Reference) | - | 1 (Reference) | - | 0.039 |
| Quintile 2 | 53/342 (15.8) | 1.31 (0.88-1.97) | 0.188 | 1.34 (0.89-2.01) | 0.161 |  |
| Quintile 3 | 47/342 (14.6) | 1.18 (0.78-1.79) | 0.444 | 1.19 (0.79-1.82) | 0.405 |  |
| Quintile 4 | 63/342 (17.5) | 1.60 (1.08-2.36) | 0.019 | 1.51 (1.02-2.25) | 0.042 |  |
| Quintile 5 | 80/343 (23.0) | 2.00 (1.37-2.92) | <0.001 | 1.84 (1.24-2.72) | 0.002 |  |
| *Excluding participants died in the first 6 months* | | | | | | |
| ^b^BA, Per 1-SD | 267/1684 (15.9) | 1.52 (1.36-1.70) | <0.001 | 1.46 (1.27-1.69) | <0.001 | 0.016 |
| BA acceleration, per 1-SD | 267/1684 (15.9) | 1.30 (1.16-1.46) | <0.001 | 1.30 (1.14-1.49) | <0.001 | 0.046 |
| Quintile 1 | 38/337 (11.3) | 1 (Reference) | - | 1 (Reference) | - | 0.038 |
| Quintile 2 | 49/337 (14.5) | 1.34 (0.88-2.05) | 0.172 | 1.38 (0.90-2.11) | 0.142 |  |
| Quintile 3 | 43/336 (12.8) | 1.22 (0.78-1.88) | 0.381 | 1.26 (0.81-1.96) | 0.297 |  |
| Quintile 4 | 59/337 (17.5) | 1.66 (1.11-2.50) | 0.015 | 1.59 (1.05-2.40) | 0.030 |  |
| Quintile 5 | 78/337 (23.1) | 2.22 (1.50-3.29) | <0.001 | 2.09 (1.39-3.15) | <0.001 |  |

Abbreviation: BA, biological age.

Model 1 is adjusted by age, race and sex. Model 2 is adjusted by the covariates in model 1 plus frailty index, smoking status, education, left ventricular ejection fraction, baseline EuroQoL, history of arterial fibrillation or flutter, history of chronic obstructive pulmonary disease, hypertension, six-minute walk distance, renal dysfunction, physical activity, use of digoxin, use of angiotensin II receptor blocker and treatment arm.

^a^ The interaction effect between treatment arm and biological age for the risk of the all-cause death is assessed by including a multiplicative interaction term (treatment arm × biological age) in model 2.

^b^ Age was not adjusted in model 1 and model 2.

Supplemental Table 7 Association of KDM-BA with death and the interaction with treatment arm by excluding the participants occur all-cause death or all-cause hospitalization in the first 6 months.

|  | **Events,**  **n (%)** | **Model 1** | ***P*** | **Model 2** | ***P*** | ^a^***P_interaction_*** |
| --- | --- | --- | --- | --- | --- | --- |
| ^b^BA, Per 1-SD | 198/1369 (14.5) | 1.49 (1.31-1.71) | <0.001 | 1.43 (1.21-1.68) | <0.001 | 0.056 |
| BA acceleration, per 1-SD | 198/1369 (14.5) | 1.26 (1.10-1.44) | 0.001 | 1.26 (1.08-1.48) | 0.003 | 0.394 |
| Quintile 1 | 29/274 (10.6) | 1 (Reference) | - | 1 (Reference) | - | 0.240 |
| Quintile 2 | 33/274 (12.0) | 1.12 (0.68-1.84) | 0.663 | 1.19 (0.72-1.97) | 0.498 |  |
| Quintile 3 | 35/273 (12.8) | 1.22 (0.75-2.00) | 0.424 | 1.32 (0.81-2.17) | 0.269 |  |
| Quintile 4 | 47/274 (17.2) | 1.64 (1.03-2.61) | 0.036 | 1.61 (1.00-2.58) | 0.048 |  |
| Quintile 5 | 54/274 (19.7) | 1.86 (1.18-2.93) | 0.008 | 1.86 (1.16-2.98) | 0.010 |  |

Abbreviation: BA, biological age.

Model 1 is adjusted by age, race and sex. Model 2 is adjusted by the covariates in model 1 plus frailty index, smoking status, education, left ventricular ejection fraction, baseline EuroQoL, history of arterial fibrillation or flutter, history of chronic obstructive pulmonary disease, hypertension, six-minute walk distance, renal dysfunction, physical activity, use of digoxin, use of angiotensin II receptor blocker and treatment arm.

^a^ The interaction effect between treatment arm and biological age for the risk of the all-cause death is assessed by including a multiplicative interaction term (treatment arm × biological age) in model 2.

^b^ Age was not adjusted in model 1 and model 2.

Supplemental Table 8 Association of KDM-BA with death and the interaction with treatment arm by including the participants aged 30 to 75.

|  | **Events,**  **n (%)** | **Model 1** | ***P*** | **Model 2** | ***P*** | ^a^***P_interaction_*** |
| --- | --- | --- | --- | --- | --- | --- |
| ^b^BA, Per 1-SD | 241/1535 (15.7) | 1.35 (1.20-1.53) | <0.001 | 1.28 (1.11-1.48) | 0.001 | 0.044 |
| BA acceleration, per 1-SD | 241/1535 (15.7) | 1.26 (1.12-1.42) | <0.001 | 1.21 (1.06-1.39) | 0.006 | 0.033 |
| Quintile 1 | 36/307 (11.7) | 1 (Reference) | - | 1 (Reference) | - | 0.023 |
| Quintile 2 | 46/307 (15.0) | 1.38 (0.89-2.14) | 0.146 | 1.38 (0.89-2.14) | 0.152 |  |
| Quintile 3 | 40/307 (13.0) | 1.15 (0.73-1.80) | 0.549 | 1.18 (0.75-1.86) | 0.467 |  |
| Quintile 4 | 53/307 (17.3) | 1.56 (1.02-2.39) | 0.040 | 1.47 (0.95-2.25) | 0.081 |  |
| Quintile 5 | 66/307 (21.5) | 1.91 (1.27-2.88) | 0.002 | 1.69 (1.11-2.59) | 0.015 |  |

Abbreviation: BA, biological age.

Model 1 is adjusted by age, race and sex. Model 2 is adjusted by the covariates in model 1 plus frailty index, smoking status, education, left ventricular ejection fraction, baseline EuroQoL, history of arterial fibrillation or flutter, history of chronic obstructive pulmonary disease, hypertension, six-minute walk distance, renal dysfunction, physical activity, use of digoxin, use of angiotensin II receptor blocker and treatment arm.

^a^ The interaction effect between treatment arm and biological age for the risk of the all-cause death is assessed by including a multiplicative interaction term (treatment arm × biological age) in model 2.

^b^ Age was not adjusted in model 1 and model 2.

Supplemental Table 9 Association of PhenoAge-BA with death and the interaction with treatment arm.

|  | **Events,**  **n (%)** | **Model 1** | ***P*** | **Model 2** | ***P*** | ^a^***P_interaction_*** |
| --- | --- | --- | --- | --- | --- | --- |
| ^b^BA, Per 1-SD | 184/1012 (18.2) | 1.40 (1.20-1.64) | <0.001 | 1.25 (1.06-1.48) | 0.009 | 0.193 |
| BA acceleration, per 1-SD | 184/1012 (18.2) | 1.12 (0.97-1.29) | 0.139 | 1.12 (0.96-1.31) | 0.156 | <0.001 |
| Quintile 1 | 37/203 (18.2) | 1 (Reference) | - | 1 (Reference) | - | 0.003 |
| Quintile 2 | 36/202 (17.8) | 0.91 (0.57-1.44) | 0.680 | 0.89 (0.56-1.42) | 0.625 |  |
| Quintile 3 | 26/202 (12.9) | 0.66 (0.40-1.09) | 0.107 | 0.72 (0.43-1.20) | 0.211 |  |
| Quintile 4 | 39/202 (19.3) | 1.01 (0.65-1.59) | 0.951 | 1.09 (0.69-1.72) | 0.705 |  |
| Quintile 5 | 46/203 (22.7) | 1.20 (0.77-1.87) | 0.424 | 1.32 (0.81-2.13) | 0.262 |  |

Abbreviation: BA, biological age.

Model 1 is adjusted by age, race and sex. Model 2 is adjusted by the covariates in model 1 plus frailty index, smoking status, education, left ventricular ejection fraction, baseline EuroQoL, history of arterial fibrillation or flutter, history of chronic obstructive pulmonary disease, hypertension, six-minute walk distance, renal dysfunction, physical activity, use of digoxin, use of angiotensin II receptor blocker and treatment arm.

^a^ The interaction effect between treatment arm and biological age for the risk of the all-cause death is assessed by including a multiplicative interaction term (treatment arm × biological age) in model 2.

^b^ Age was not adjusted in model 1 and model 2.

Supplemental Table 10 Subgroup analysis of the association between BA and death stratified by sex.

|  | **Events,**  **n (%)** | **HR (95%CI)** | ***P*** | ^a^***P_interaction_*** | ^b^***P_interaction_*** |
| --- | --- | --- | --- | --- | --- |
| *Male* | | | | | |
| ^c^BA, Per 1-SD | 240/1260 (19.0) | 1.36 (1.18-1.57) | <0.001 | 0.020 | 0.479 |
| BA acceleration, per 1-SD | 240/1260 (19.0) | 1.23 (1.08-1.41) | 0.002 | 0.037 | 0.689 |
| Quintile 1 | 37/252 (14.7) | 1 (Reference) | - | 0.093 | 0.937 |
| Quintile 2 | 43/252 (17.1) | 1.17 (0.75-1.82) | 0.486 |  |  |
| Quintile 3 | 47/252 (18.7) | 1.36 (0.88-2.11) | 0.162 |  |  |
| Quintile 4 | 45/252 (17.9) | 1.31 (0.84-2.04) | 0.228 |  |  |
| Quintile 5 | 68/252 (27.0) | 1.82 (1.19-2.78) | 0.006 |  |  |
| *Female* | | | | | |
| ^b^BA, Per 1-SD | 61/472 (12.9) | 1.60 (1.09-2.35) | 0.017 | 0.300 | 0.479 |
| BA acceleration, per 1-SD | 61/472 (12.9) | 1.35 (0.91-1.99) | 0.134 | 0.244 | 0.689 |
| Quintile 1 | 8/95 (8.4) | 1 (Reference) | - | 0.074 | 0.937 |
| Quintile 2 | 14/94 (14.9) | 2.10 (0.86-5.12) | 0.105 |  |  |
| Quintile 3 | 9/94 (9.6) | 1.34 (0.50-3.59) | 0.567 |  |  |
| Quintile 4 | 15/94 (16.0) | 2.31 (0.94-5.68) | 0.067 |  |  |
| Quintile 5 | 15/95 (15.8) | 1.62 (0.63-4.15) | 0.316 |  |  |

Abbreviation: BA, biological age.

The model is adjusted by age, race, frailty index, smoking status, education, left ventricular ejection fraction, baseline EuroQoL, history of arterial fibrillation or flutter, history of chronic obstructive pulmonary disease, hypertension, six-minute walk distance, renal dysfunction, physical activity, use of digoxin, use of angiotensin II receptor blocker and treatment arm.

^a^ The interaction effect between treatment arm and biological age for the risk of the all-cause death is assessed by including a multiplicative interaction term (treatment arm × biological age) in the model.

^b^ The P for interaction between sex × BA using the overall cohort.

^c^ Age was not adjusted in the model.

Supplemental Figure 1 Flowchart for the selection of study participants


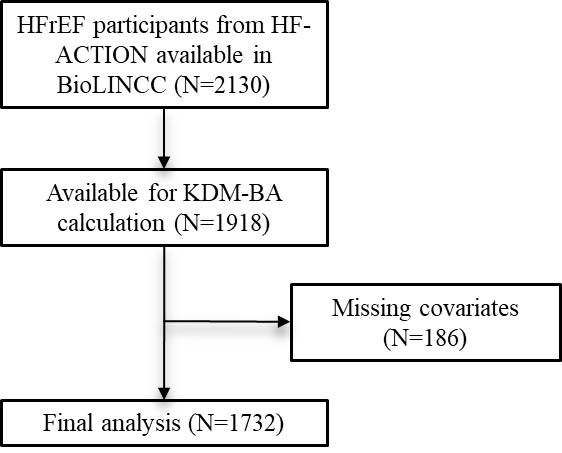


Supplemental Figure 2 Correlation matrix between KDM-BA biomarkers and age


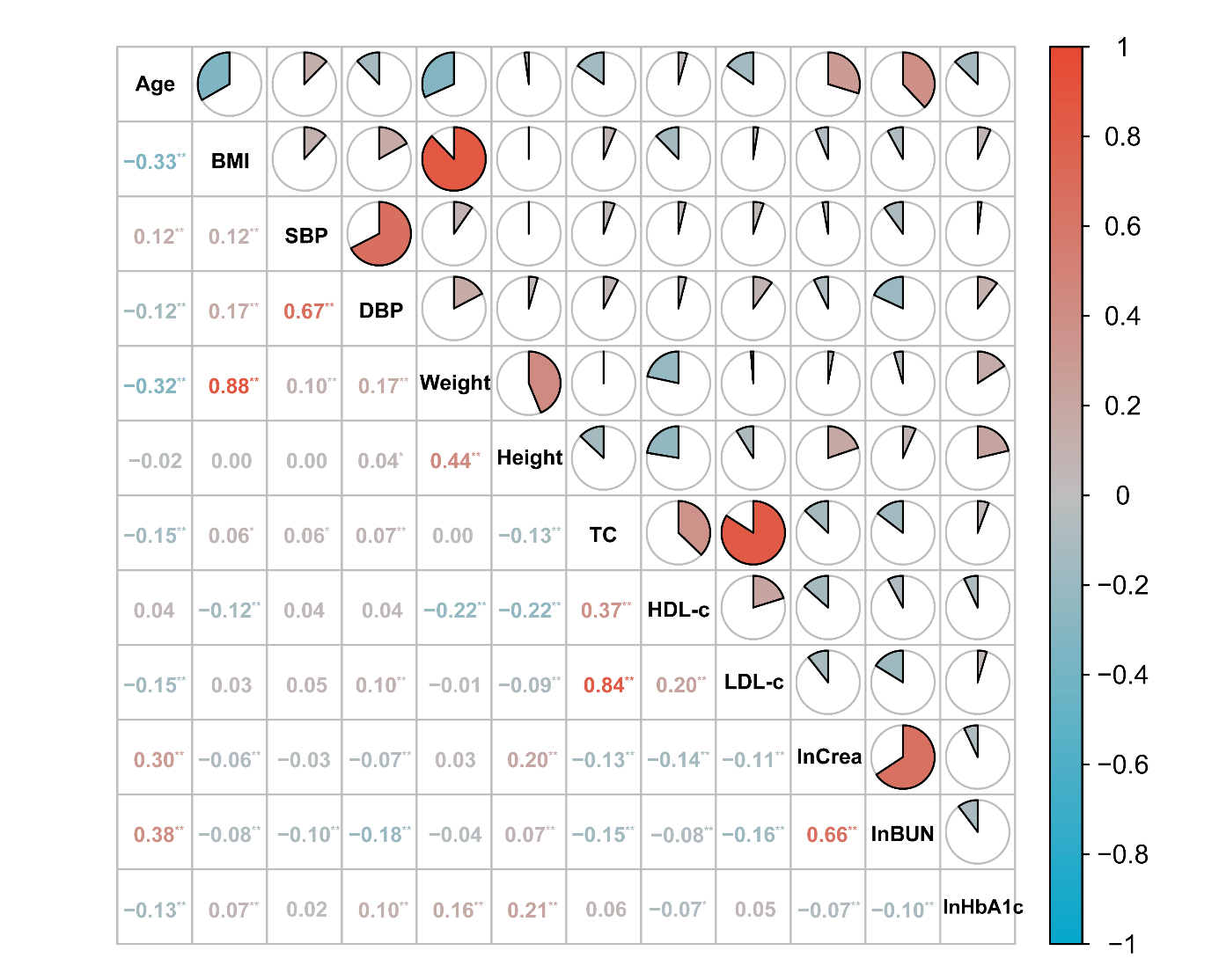


Abbreviations: BMI indicates body mass index; SBP, systolic blood pressure; DBP, diastolic blood pressure; BUN, blood urea nitrogen; HbA1c, glycated haemoglobin; Crea, Creatinine; TC, Total cholesterol; HDL-c, high density lipoprotein cholesterol; LDL-c, low density lipoprotein cholesterol.

Supplemental Figure 3 Correlation between KDM-BA and chronological age


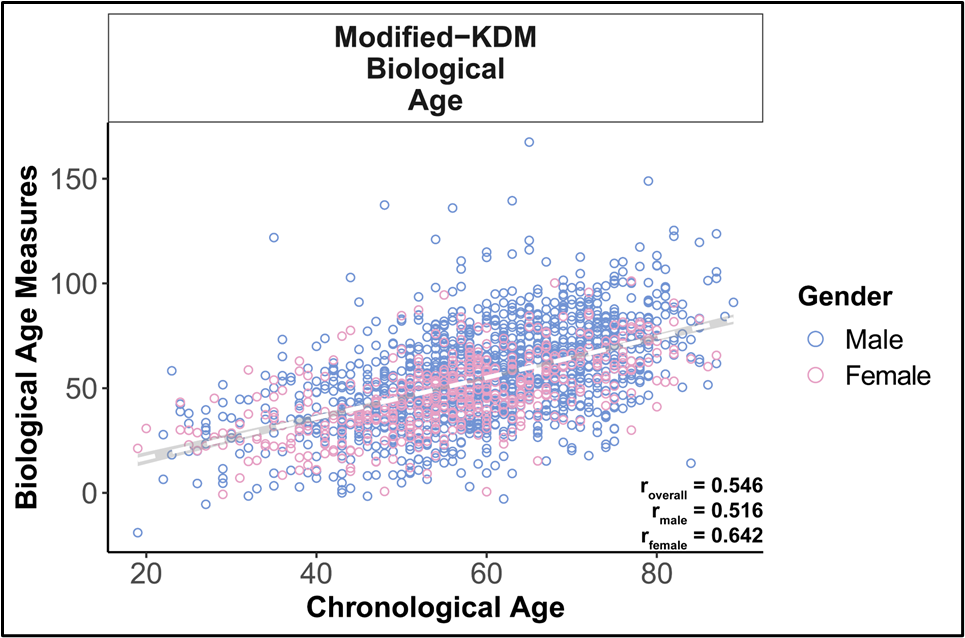


Supplemental Figure 4 Correlation between KDM-BA acceleration and chronological age


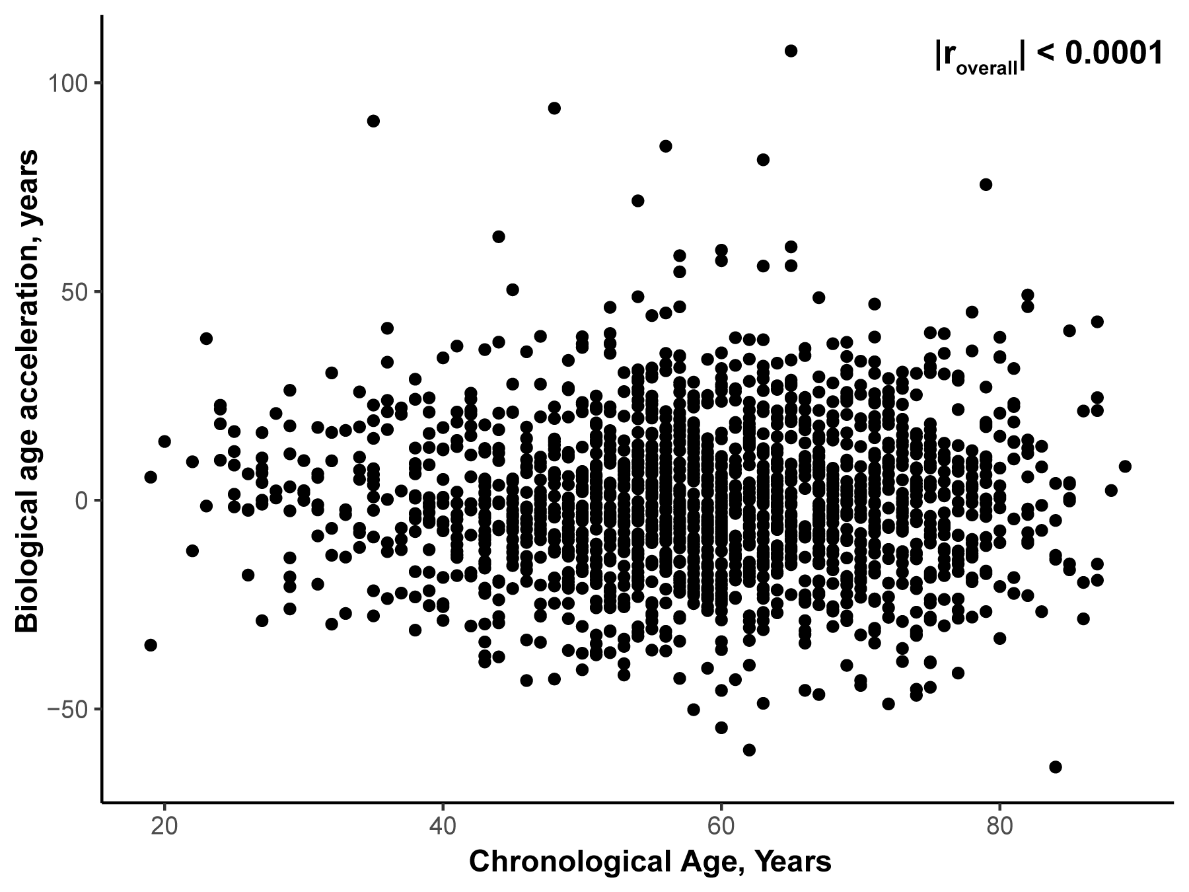


Supplemental Figure 5 Primary and secondary outcomes stratified by biological age acceleration in the overall cohort


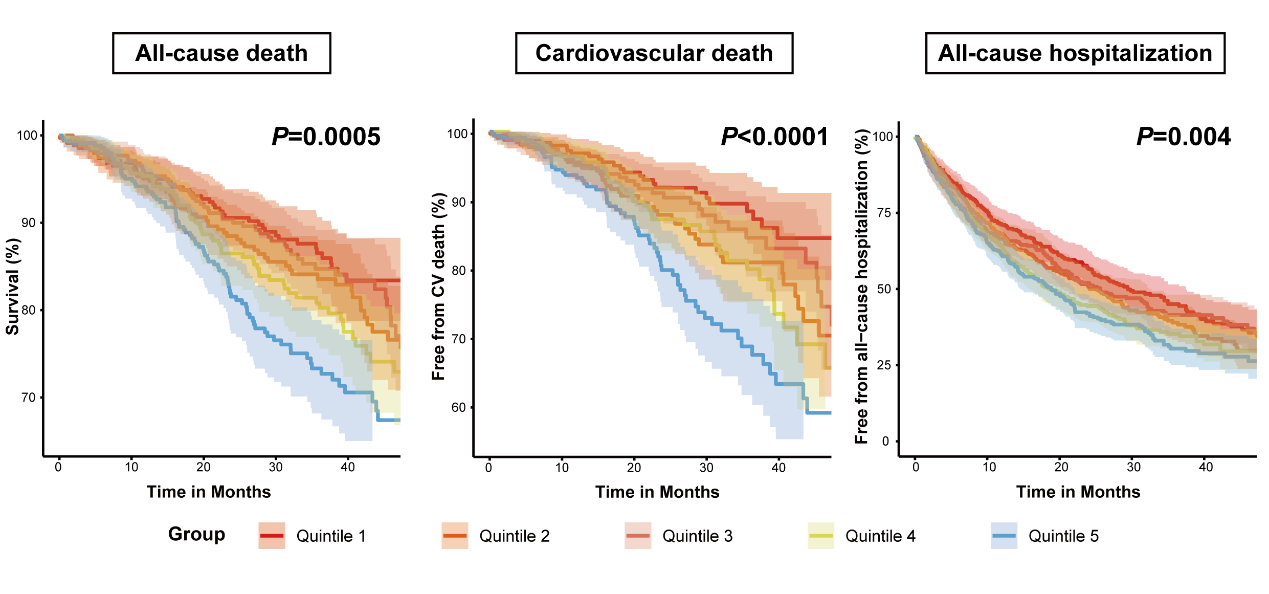


Supplemental Figure 6 Incidence of free from primary and secondary outcomes stratified by biological age acceleration


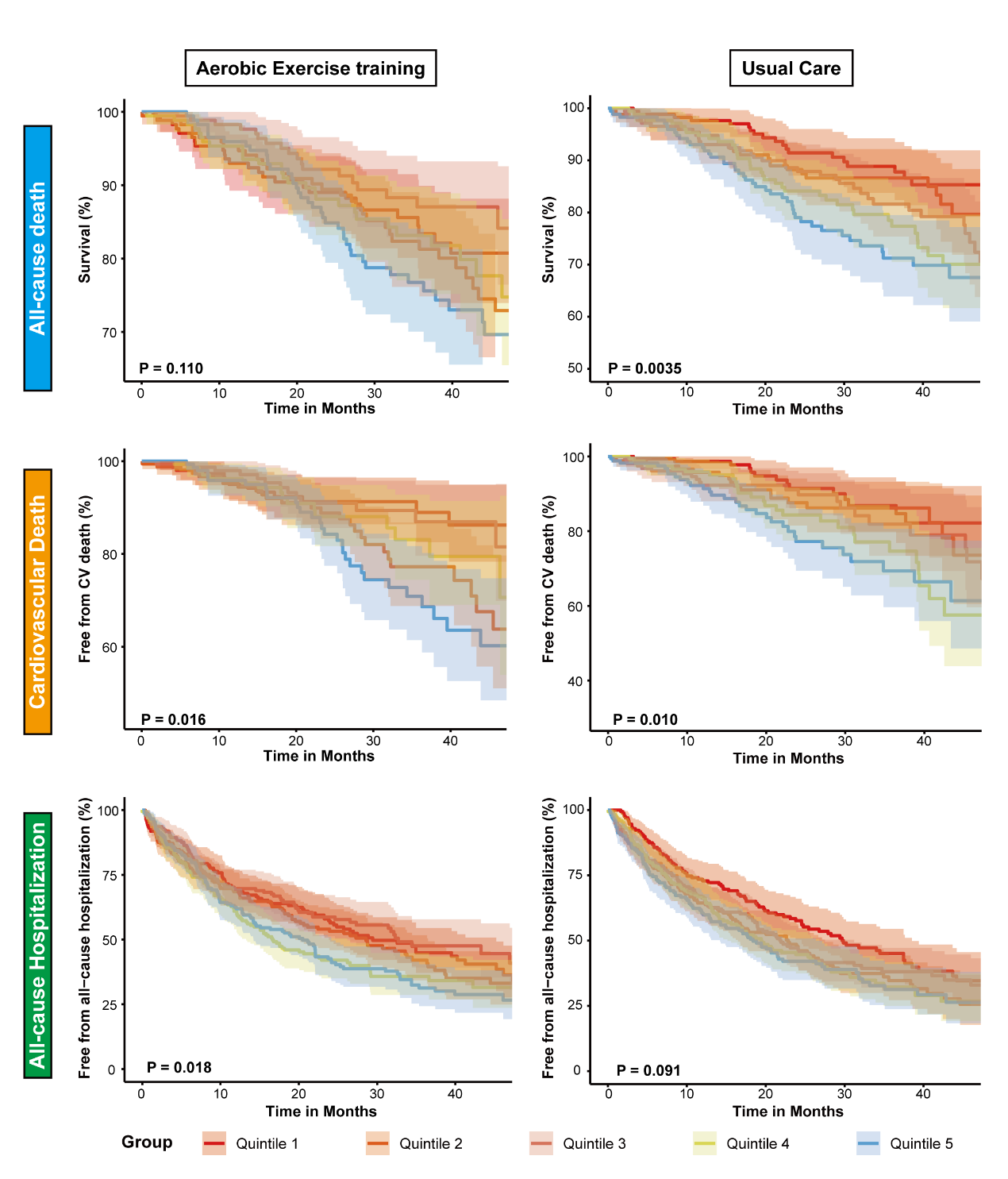


Abbreviations: CV, cardiovascular.

Supplemental Figure 7 The best fitting models for relationships of KDM biological age with risk of secondary outcomes stratified by treatment arms


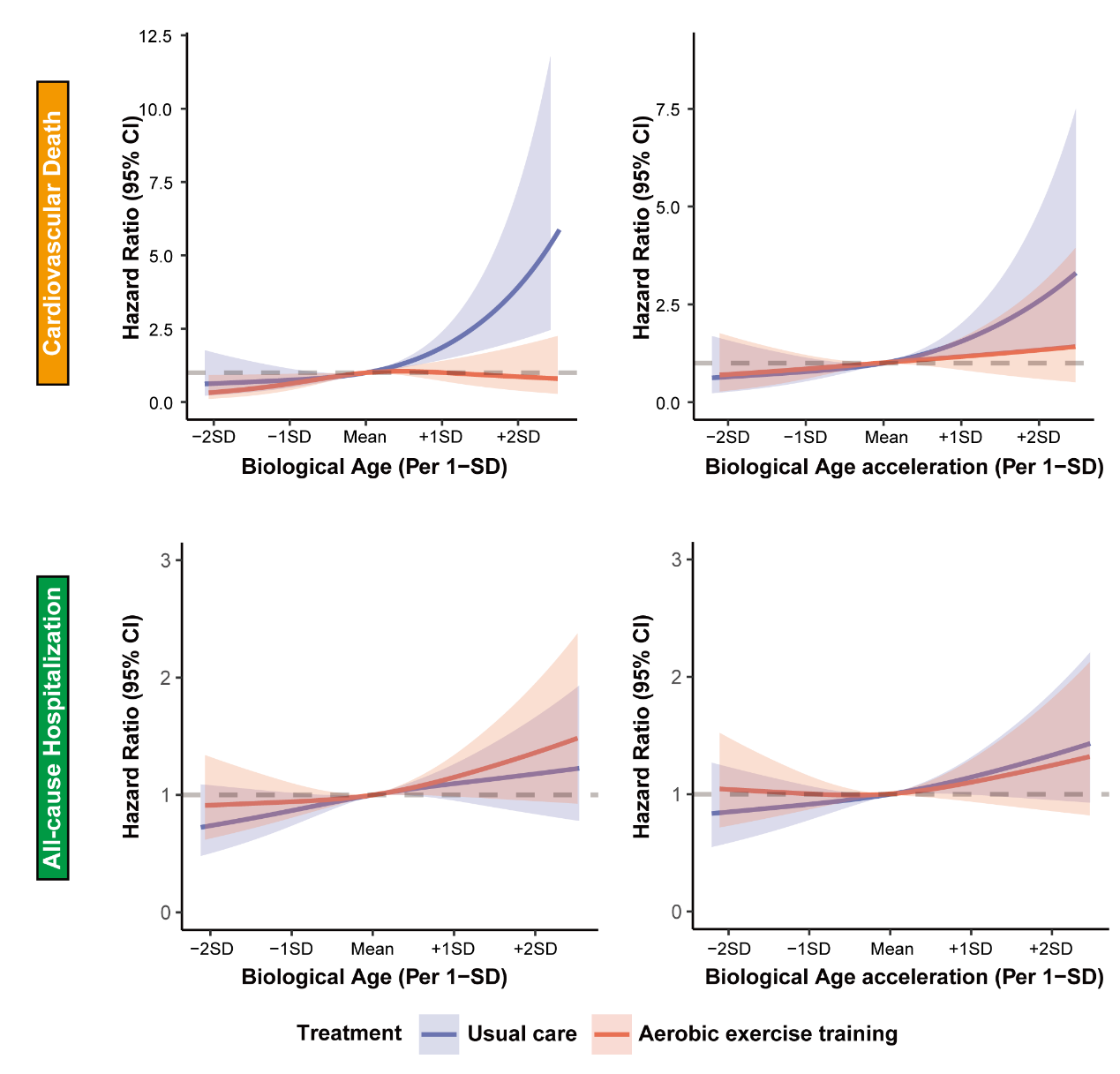


Abbreviations: CV, cardiovascular; SD, standard deviation.

Solid line indicates the point estimation, ribbons indicate the 95% confidence intervals, and the grey dash line indicates the reference line (y=1). Restricted cubic spline regression model by three knots (10th, 50th, and 90th) were constructed for participants in exercise (EXE) and control (CON) group separately for risk of all-cause and cardiovascular death with adjustment for the same covariates. Adjusted covariates include race, sex, frailty index, smoking status, education, left ventricular ejection fraction, baseline EuroQoL, history of arterial fibrillation or flutter, history of chronic obstructive pulmonary disease, hypertension, six-minute walk distance, renal dysfunction, physical activity, use of digoxin, use of angiotensin II receptor blocker. Age was additionally adjusted in models of biological age acceleration.

Supplemental Figure 8 The mediating proportion of biological age on the association between physical activity, aerobic exercise and all-cause death


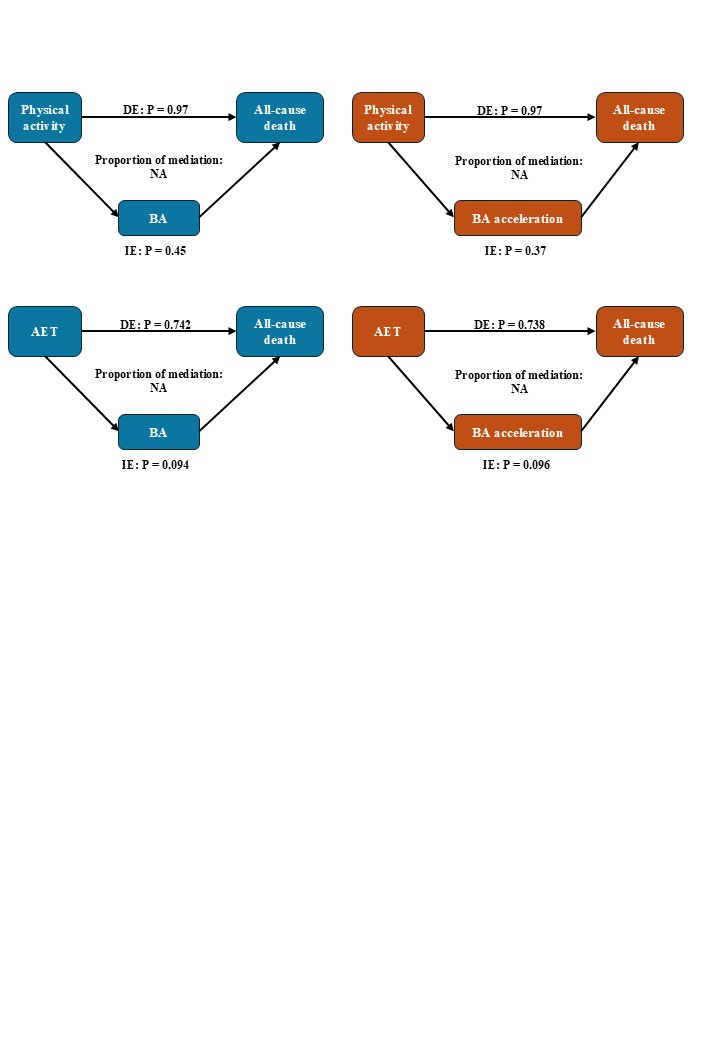


Abbreviations: BA, biological age; AET, aerobic exercise training; DE, direct effect； IE, indirect effect.

Adjusted covariates include race, sex, frailty index, smoking status, education, left ventricular ejection fraction, baseline EuroQoL, history of arterial fibrillation or flutter, history of chronic obstructive pulmonary disease, hypertension, six-minute walk distance, renal dysfunction, physical activity, use of digoxin, use of angiotensin II receptor blocker. Age was additionally adjusted in models of biological age acceleration.
